# Engendered Expressions of Anxiety: Men’s Emotional Communications With Women and Other Men

**DOI:** 10.3389/fsoc.2021.697356

**Published:** 2021-06-29

**Authors:** Brendan Gough, Steven Robertson, Hannah Luck

**Affiliations:** Leeds School of Social Sciences, Leeds Beckett University, Leeds, United Kingdom

**Keywords:** anxiety, emotional communication, men, masculinity, interviews, thematic analysis, men and masculinity

## Abstract

While the contemporary therapeutic discourse inveigles us to talk about our personal problems, a countervailing neo-liberal healthist discourse, aligning with conventional masculinity norms, presumes that we will manage any issues independently. This discursive tension can be difficult to navigate, especially for men confronted with still powerful traditional expectations around masculinity (e.g., self-reliance; personal control; restricted emotionality). Although qualitative research has examined how men negotiate masculinities with respect to depression, to date there has been scant attention focused on men experiencing anxiety. This article reports on an interview study with men, some with anxiety diagnoses and some without (*N* = 17). Thematic analysis highlights that participants can and do talk about their anxieties, most readily with significant women in their lives (e.g., partners; mothers)–although this is not always straightforward. Talking to other men was more fraught, and while participants were wary of sharing problems with male friends, or signaled issues indirectly, they also highlighted situations where they would open up e.g., workspaces where they felt safe; with best friends. Those who had gone through a therapeutic process over many years tended to me more comfortable talking to others, male or female, about their mental health–and were also keen to other support to others where they could. Our analysis suggests that despite stereotypical notions of silent, self-contained men, there are many contexts where men may feel comfortable sharing their stories of pain and suffering. This chimes with wider cultural changes and the reported experiences of some mental health initiatives.

## Introduction

According to the 2013 Global Burden of Disease study, anxiety is the second most commonly diagnosed mental health problem worldwide (Vos et al., 2013), with approximately 264 million people thought to suffer from anxiety globally (World Health Organization (WHO), 2017). While globally, depression remains the most commonly identified mental health problem (World Health Organization (WHO), 2018), other statistics suggest comorbid diagnoses of anxiety and depression to be the most commonly diagnosed condition in the United Kingdom (NICE, 2011), with anxiety disorders representing the second most commonly diagnosed mental health problem (McManus et al., 2016). Either way, it is clear that anxiety affects a large number of individuals living in the United Kingdom (and beyond).

Gender differences in the statistics relating to mental health are widely recognised, with women understood to be twice as likely to be diagnosed with an anxiety disorder or depression throughout their lives (Remes et al., 2016). Researchers have suggested that the lower prevalence rates of common mental health problems in men do not mean that men experience depression or anxiety less overall. Rather, their expressions of symptoms are constrained by hegemonic masculinities that privilege practices such as self-reliance and restrictive emotionality (Addis and Mahalik, 2003; Mahalik and Rochlen, 2006; Robertson et al., 2016). Given the association between depression and suicide (Zhang and Li, 2013; Luo et al., 2016), it is perhaps unsurprising that the overwhelming majority of research has focused on how men experience and manage depression (e.g., Oliffe et al., 2016; Lee et al., 2017; Seidler et al., 2018). This focus has meant that other important common mental health problems, particularly anxiety, have been relatively unexplored in gender and mental health research. Recent research on changing masculinities, emotion and vulnerability suggests that, despite persistent stereotypes of emotionally repressed men, there are circumstances where some men may be willing to talk about their fears and anxieties. For example, certain online environments like discussion forums can enable men to (anonymously) share their distress and receive support from peers with similar experiences e.g., depression, infertility (e.g., Gough, 2016; Hanna and Gough, 2016). In fact, recent research by Drioli-Phillips et al. (2020a), Drioli-Phillips et al. (2020b) has examined how men talk to each other about anxiety online and highlights the importance of presenting credible accounts, with anxiety characterised as loss of control.

Mental health support online can also target particular communities of men e.g., from ethnic and sexual minority groups (e.g., the British Punjabi community: http://www.taraki.uk/male-mental-health). Offline, a range of community initiatives targeting men, often activity-based, have been implemented (see Robertson et al. 2016), perhaps the best known being the “Men’s Sheds” initiatives bringing older isolated men (and increasingly other groups of men) together to work on practical projects and develop bonds (e.g., Ballinger et al., 2009). Here, the emphasis is on indirect, “shoulder-to-shoulder” support in a friendly environment. But there are also some initiatives encouraging men to directly share their mental health problems with other men, face-to-face (e.g., Andy’s Man Club: https://andysmanclub.co.uk/)–an interesting development considering men’s supposed horror of “just talking” about their emotional difficulties and perhaps signaling a shift toward a softer, more caring and emotionally intelligent masculinity.

Aside from seeking and/or receiving support from peers within designated spaces and programmes, literature on (heterosexual) interpersonal relationships suggest that men may be somewhat comfortable disclosing to female partners, within limits (e.g., Holmes, 2015). In a study exploring the interplay between discourses of “big boys don’t cry” and “it’s good to talk”, McQueen (2017) found tensions for men surrounding the need to disclose in intimate relationships and a gendered preference toward remaining self-reliant. With some younger men, mothers may be pivotal in enabling self-disclosure (Wirback et al., 2018). Robertson (2007), however, reminds us of the dangers of over-emphasising the role that significant female others may have, arguing that placing the burden of men’s emotional well-being on women removes responsibility from men in effecting positive changes for themselves. As for men’s friendships with other men, the literature largely focuses on notions of competition, policing and social comparison whereby traditional masculinities are enacted and perpetuated (e.g., Kimmel, 1994). However, recent theory and research points to a loosening of such expectations. If we consider the body of research underlying Anderson’s inclusive masculinity theory, for example, it is reported that young, middle-class, white men are engaging in more emotionally expressive forms of friendship (see Anderson, 2008; Anderson and McGuire, 2010; Adams, 2011). In terms of mental health issues, however, little is known about how men might expose their difficulties to male friends and peers.

Recognising the gap in research on men’s anxiety, this article seeks to understand more about how men construct and negotiate everyday and significant anxiety experiences. In particular, we were interested in men’s accounts of help-seeking and self-disclosure in the context of anxiety, and in identifying situations and relationships where men felt most comfortable sharing their worries.

## Materials and Methods

Initially, our intended focus was on men with a current diagnosis of Generalised Anxiety Disorder (GAD). Considered a chronic disorder (Yonkers et al. 2000), GAD is generally characterised by excessive, persistent and disruptive worries that are not restricted to particular events or situations (Lader, 2015), as well as other physically disruptive symptoms (Cuijpers et al., 2014). However, after searching for literature pertaining to men and anxiety, there were few results. There also appeared to be no direct or sustained gendered discussion of anxiety, either as an emotion in its own right or as a mental health issue. GAD is very rarely diagnosed alone, most commonly being diagnosed in relation to a wide spectrum of other disorders (Wittchen et al., 1994), such as panic disorder and social anxiety (Yonkers et al., 1996), bipolar disorder (Pavlova et al., 2015) and major depressive disorders (Spitzer et al., 2006). As such, it was decided to open up the inclusion criteria to participants with a mental health diagnosis in order to include a wide range of anxiety disorders and comorbidity with depression. The study therefore addressed how anxiety is managed in everyday (non-pathologised) contexts as well as in relation to GAD, thereby garnering a rounded view of men’s accounts of anxiety and any associated help-seeking practices.

The work was undertaken in three phases: the first was a qualitative survey on anxiety experiences distributed to men and women; the second conducted interviews with men without a formal diagnosis of anxiety; the third phase consisted of interviews with men who did have a formal diagnosis of anxiety. We report here on phases two and three (NB The splitting of the latter two phases into diagnosed/not-diagnosed was not used to argue for necessary differences in how anxiety could be experienced or managed. Rather, it was used to aid the research process in terms of gaining access to participants and we recognise that we must not assume that experiences of anxiety are necessarily more severe for those with a diagnosis compared to those without). Before the study commenced recruitment and data collection, ethical approval was obtained from (blinded for peer review) university ethics committee.

### Sampling and Recruitment

The recruitment of men for phase two was facilitated through a question on the phase one survey asking if they would provisionally be willing to participate in an interview. The survey focused on everyday experiences of anxiety for men and women, and was advertised via the university, partner organisations and social media (*N* = 104; 56 women; 48 men). Ten participants were recruited to this study through this phase two process; none reported any current anxiety or mental health diagnosis. For phase 3, United Kingdom charities supporting people with anxiety were contacted, and a recruitment call was placed on Twitter. This recruitment call process was repeated on two further occasions. The criteria set down for phase three required that all participants were currently diagnosed with an anxiety disorder, with allowances made for comorbidities with other affective disorders, such as other anxiety disorders and depression. Note: we relied on self-report rather than medical records regarding diagnosis and did not specify candidate symptoms or duration of illness. The final number of participants recruited to phase three through this process was seven, with two reporting co-morbid depression, two citing co-morbid PTSD and one mentioning a former diagnosis for OCD.

For both phases reported here, potential participants were provided with an information sheet regarding the research project. To navigate any potential distress caused by the discussion of “sensitive topics”, participants were informed that they were able to skip questions or withdraw from the interview altogether at any point. Contact information for anxiety helplines were also provided on the information sheet.

Brief biographical vignettes for the 17 participants ultimately recruited to phase two and phase three are provided in Table 1. To help ensure anonymity participants assigned themselves names (pseudonyms). Quotes used later in this paper include these self-assigned names but also include “P2” or “P3” in brackets to identify the phase that they were recruited to.

**TABLE 1:** : Participants.

Phase 2 (*N* = 10)	Phase 3 (*N* = 7)
P2.1 Malcolm 55 years old, white British and married with one adult child with cystic fibrosis. He has a high-powered managerial job in the banking industry	P3.1 Russell 38 years old, Asian British, single with no children. Diagnosed with PTSD, anxiety, depression, and hypersensitivity after a traumatic event in his teenage years. He has a part-time job and works with raising mental health awareness in his area
P2.2 Matthew 58 years old, Black British, heterosexual and divorced. He has two adult children and holds a managerial position in a governmental agency	P3.2 Frank 49 years old, white British, single with no children. He was diagnosed with social anxiety two weeks prior to his interview but acknowledged that anxiety has been a presence in his life since childhood. He has a full-time job as a chef
P2.3 Isaac 62 years old, white British, heterosexual and divorced. He has two stepchildren, one adopted child and was a foster carer to three more children. He is a semi-retired property developer-turned academic. He hopes to pursue a Ph.D. over the coming years in psychology	P3.3 Dylan 49 years old, white British/Irish divorced with one adult child. Diagnosed with acute anxiety and depression, he also has an extensive history of self-harm and substance abuse. He works full-time as an educator and campaigner for mental health issues
P2.4 Thomas 27 years old, white British, heterosexual and in a long-term relationship. He does not have children and is a freelancer in the arts and entertainment industry	P3.4 Ralph 54 years old, white British, unspecified marital status and no children. Diagnosed with anxiety, he works as a librarian. He has previously been a music blogger and now runs a mindfulness website
P2.5 Lincoln 27 years old, Asian British, heterosexual and single with no children. He is studying for his second masters, after completing his first one in gender studies. Currently spends his time split between West London and Oslo. He received a diagnosis of anxiety and depression in the months following his interview	P3.5 Patrick 44 years old, white British married with no children. Diagnosed with PTSD following a car crash. He has an extensive medical history in both physical and mental health spheres
P2.6 Fabien 67 years old, white British, heterosexual man and divorced. He has adult children and is a retired mental health nurse. Despite never receiving a diagnosis of anxiety, he has experienced acute anxiety at certain points of his life	P3.6 Christopher Late twenties, white British, in a long-term relationship with no children. Diagnosed with panic disorder and social anxiety. He has also received treatment for OCD and agoraphobia in the past. He works as a post-doctoral scientist
P2.7 Gareth 46 years old, white British, heterosexual and married with two children. He holds a high-level position in project management	P3.7 Leo 33 years old, white British, long-term partner and no children. Diagnosed with panic disorder (with a specific phobia of fainting in public). He works as a full-time firefighter and is active in social media conversations surrounding men’s mental health
P2.8 Saleem in his thirties, Zimbabwean national, living in the United Kingdom. Heterosexual and married with one young child. He works as an engineer and has an active Muslim faith	
P2.9 Jack 39 years old, white British, heterosexual and married with two young children. He holds a managerial position at a bio-engineering firm in London and had recently been signed off work due to stress	
P2.10 Henry 25 years old, Welsh, heterosexual and single with no children. He recently graduated from his undergraduate degree in Liverpool and is an avid artist. He had experienced a phobia of water as a child	

### Data Collection

Once recruited, semi-structured interviews were conducted in 2017 with participants sequentially across the two phases. This is a well-established method which allows researchers to explore predetermined topics while also allowing the participants to expand upon their answers and experiences (Wahyuni, 2012). After setting participants at ease by asking them to say a little about themselves, a broad opening question was asked: “Can you tell me about any experiences you’ve had with anxiety?” The topic guide subsequently included question areas around “doctors experiences”, “medication” and “recognising anxiety”. The majority of the face-to-face interviews were conducted at the university or at the participant’s workplace depending on their preference.

It was anticipated that some participants (mainly in phase three) may not be able or willing to conduct the interviews in person and so Skype was offered as an alternative in both phases. Two of the phase two interviews and four of the phase three interviews were conducted via Skype. Interviews lasted 45–60 min in phase two and 60–120 min in phase three. Many of the phase three participants had been involved with varying levels of health care services, some over many years, and so appeared to be more comfortable with talking about their experiences with anxiety. The interviews in both phase two and three were recorded and transcribed by one of the authors and interview transcripts were returned to the participants to read over and make any amendments before moving forward into data analysis.

### Biographical Note on Conducting the Interviews

Many of the participants appeared to expect a researcher in a white lab coat, with a clipboard and pen, ready to record their responses on a sheet of paper. As such, many appeared surprised to be greeted by a short, young lady with glasses too large for her face and without a clipboard or pen in sight. Despite a usually hesitant start, many of the participants relaxed significantly into the interview process after the first “springboard” question had been asked, and most interviews progressed with a conversational style. However, the two youngest participants in phase two appeared to remain uncomfortable throughout the interview process and required more input and prompting from the researcher-it should be noted that these interviews were conducted over Skype and so the lack of “in person” interaction may also have impacted how comfortable they felt throughout the interview process.

### Analysis

As a method that can allow for both the summarisation of key features in a large body of data while also offering opportunities for thick descriptions of datasets (Braun and Clarke, 2006), thematic analysis was chosen. Thematic analysis places primacy on the active role that the researchers play in identifying patterns and in making decisions about which patterns best address the research aim. Importantly, it also allows for the identification of semantic (descriptive) and latent (hidden, implied) meaning within the data (Braun and Clarke, 2006). The six steps for thematic analysis, developed by Braun and Clarke (2006), was used as a guide throughout the analysis of the data, moving from data immersion and preliminary coding through to forming candidate themes and revising, refining and writing-up these themes. Initial coding and early categorising was undertaken by the lead researcher and amended in discussion with the team. Data collection continued, in both phases, until data saturation was reached and no new categories were being developed. While the numbers recruited to each of the two phases seem small, numerous studies have shown that the majority of themes (70–99%) are identified from the first six to ten interviews (Guest et al., 2020) and this was the case in this study. Further refinement of categories and development of themes was completed through research team discussions.

## Results

While the therapeutic discourse was invoked by our interviewees, this was complicated and constrained by neo-liberal and masculinity discourses privileging (male) resilience and responsibility and disavowing emotional talk. In the interviews, emotional communication and self-disclosure tended to be situated mostly within close romantic relationships, although in all-male contexts some (indirect) emotional talk was deemed possible. Analysis therefore generated two overarching themes: “Engendered anxiety disclosures with women”, and “Calculations and covert communications with other men”. These themes highlight not only how men are able and willing to engage in forms of emotional talk, but they also underline the importance of social relationships in supporting men to manage mental health issues.

### Engendered Anxiety Disclosures With Women

Throughout the research, accompanying the perception that (other) men do not speak openly about their emotions, was the idea that women are more emotionally aware and open than men. Consequently, many of the participants highlighted a preference toward talking to female friends, family members or health professionals when dealing with anxiety:

I actually find ladies have more empathy, more engaging. I’m gonna be quite honest with you, if you were a guy, I probably wouldn’t have accepted your invitation (to be interviewed) so... I just couldn’t open up as much as you know... if I was worrying and wanted to talk to someone, I would always go to a woman. (Patrick, P3)

Romantic partners, in particular, were singled out as especially adept at eliciting disclosure, as previous research has noted (e.g., Holmes, 2015; McQueen, 2017). Fabien (P2), who described himself as having a *“moderate anxiety complaint”*, mentioned that his anxiety levels notably diminished after he married. Similarly, Matthew (P3) stressed the efficacy of talking to romantic partners in helping to alleviate anxiety: *“When I was with my long-term partner, there you talk about everything because it was what you did”*.

Indeed, many of the participants explained that emotional talk had been positioned as a non-negotiable part of their close relationships. Saleem (P2) explained it in this way:

I: Can you explain to me how you started to talk more about your emotions?
S: Well (laughing) my mum used to shout at me for not talking like “can you please tell me what you’re feeling” and then I think yeah... when I got married and my wife explained to me that, “listen, it’s not a burden. Just explain your feelings and all the rest...” and that’s when I started chatting.

In this sense, disclosures of anxiety are *engendered*–in part engineered by significant female others, including mothers (see also Wirback et al., 2018). Christopher (P3) also refers to his mother and female partner who were expressing concern about his reticence during a difficult period and their insistence on him opening up:

I got very withdrawn again and my mum and my girlfriend got very worried, which is completely reasonable, but I do just tend to completely clam up... Just don’t talk about it, internalise it. Um, they both let me know they were not happy with that arrangement so, I do try and talk about it more.

Clearly, for Christopher, emotional communication is an ongoing project with which he continues to struggle. Beyond the expectation that men will engage in emotional talk within romantic relationships, it appeared that the ability of female friends or family members to observe, perceive and enquire about a close male relative or partner’s emotional well-being was similarly important. In reflecting on his emotional talking practices with a close female friend, Christopher noted:

I, myself, have wondered why I am happy to speak to Diane I guess … I’m happy to answer the questions if people ask them. If people, ask me the right questions... for the most part anyway. If I’m having a really bad day, I just want to be left alone...

In outlining the importance of being asked the “right questions”, Christopher’s response demonstrates the centrality of relationships in encouraging (or constraining) types of emotional disclosure that men will use. It is also important to highlight that Christopher’s willingness to speak to Diane is influenced by factors besides her gender. In exploring their friendship, he explained that she is both a clinical psychologist and personally suffers from anxiety. As a result, Christopher explained: *“she’s just very open and forward-thinking in her attitude toward anxiety, as you’d hope for somebody in her position”*. As will be demonstrated later, establishing trust and shared ground can encourage more open forms of emotional talk in the presence of other men. The importance of being asked “the right questions” was similarly reported by Fabien:

I have a partner who I have been with for three years... she does say “are you alright” and things like that from time to time. I think I went through a bit of a period last year when I was wrestling with all sorts of things, probably to do with the relationship-my future with her and so on which, I didn’t particularly want to talk about. I suppose I could’ve been led into it if she’d have... got her own sort of talent, therapeutic talent and sort of dragged it out of me a bit more skilfully.

In asserting that he could have been “led into” an open discussion about emotions if it had been “dragged out” of him, Fabien highlights again the importance of relationships in helping to encourage emotional openness and the development of new anxiety management practices from someone initially reluctant to do so.

However, while the majority of participants noted that they would be more likely to view female partners, relatives and friends as more understanding of mental health issues, engaging in open emotional talk with women was not without its complications. Some participants also reported feeling frustrated when engaging in emotional topics with their partners or close relatives, as Malcolm (P2) highlighted:

M: I think there’s stuff that perhaps over the years we should’ve shared more deeply at an earlier stage...
I: Do you know the reason why you haven’t discussed them?
M: Yeah... You know, it’s going to be a half-hour conversation and there’s something you want to watch on the telly in 10 min (laughing)... I was going to say you need to be precise in your language but, sometimes, you can be too precise in your language and you just need... (pause). Perhaps it’s me but, it seems that quite a lot of what I say gets misunderstood (laughing)...

Feelings of being misunderstood within relationships, or lacking the vocabulary with which to adequately describe or relate to emotional conversations, were reported by six participants, reminding us that close relationships do not automatically generate therapeutic conversations. Johnson et al. (2012) use the phrase “guarded vulnerability” to convey how their male interviewees characterised self-disclosure to partners in the context of depression, and it seems apt in relation to anxiety here. So, the social webs surrounding individuals are complex, and in significant personal relationships the emotions of the “other” may impede the development of sensitive exchanges.

The notion that direct and open emotional conversations did not feel “natural”, even when engaging with women (mothers, wives, female friends or female health professionals), was reported by other participants. After experiencing his breakdown, Jack highlighted the development of his talking practices with his wife:

We do talk more about things and it feels a little bit forced almost … you know, because you sort of learn a technique … yeah we just talk more about things and it’s just a bit more natural. It’s probably a long way still to go but when you look back and think where I was then, it’s quite different now.

In outlining the need to learn the “technique” of emotional talk, Jack’s experiences are particularly interesting and link to Malcolm’s previously noted feelings of frustration. Holmes (2015) similarly highlighted the need for men to “learn” how to engage in verbal forms of support in distance relationships, with her participants reporting their initial attempts as difficult or cumbersome. Sometimes it is too difficult. Lincoln linked this issue to the dissolution of his relationship with his girlfriend, conceding that he has work to do to develop his emotional communication skills. Indeed, there are interventions which specifically target men who find emotional expression difficult, such as “alexihtymia reduction therapy”–alexithymia referring to an inability to recognise or describe emotions (Levant et al., 2009).

### Calculations and Covert Communications With Other Men

Despite traditional understandings of homosocial interactions being policed and constrained by traditional masculinity norms (e.g., restricted emotionality; competitiveness; strength; e.g., Kimmel, 1994), with particular criticism reserved for men who try to talk about emotions (Felmlee et al., 2012), there is evidence that men can and do talk to each other about personal problems, albeit mostly indirectly (Lefkowich et al., 2017). However, this often requires complex negotiations around presentations of their male identity (Mackenzie et al., 2017). In this section, we identify the ways in which our participants navigate between such traditional expectations and more contemporary injunctions toward help-seeking and self-disclosure.

The notion that men do not openly talk about their emotions with other men echoed throughout the research. Nonetheless, the policing (or fear of being policed) of men’s emotional expressivity within all-male settings did not necessarily stop the participants from (partly) engaging in emotional talk with their friends, and the forms that emotional talking took were heterogeneous. Even Dylan, who outlined struggling with opening up to men in the past, now regularly engages with other male service users in direct and open discussions about mental health and emotional wellbeing. Indeed, several participants drew attention to the ways that they may navigate their emotional engagements with friends:

There’s always a limit to what you talk about. There’s never a full-blown conversation about feelings and emotions but there always is in some sort of form... you can speak openly more to some than others. (Henry, P2).

Henry’s response highlights that there can be emotional communication between men, albeit limited, and that the extent of this may vary by relationship and context. With much of the literature stressing that men are more likely to engage in instrumental friendships (e.g., Robertson and Monaghan, 2012), it is easy to lose sight that men (and women) have many different types of friendships (see Frey et al., 2016).

Fabien (P2) neatly captures the calculations, potential risks and benefits of emotional talk with male friends:

F: I think we all keep an eye on each other in a very covert way. We know each other well enough and if there’s two of the three of us are in conversation and we mention the other, we’ll say “well he’s not so good at the moment” or “something’s happening” or maybe... “is he ok?” ... You know, we’ll go into that sort of territory, gossiping if you want but yeah... it’s concern-concern for the other. But that’s the way it happens for men, it’s covert. It’s in friendships or in a social context where um, you know, there’s a pairing or a small group that are genuinely, long-term concerned friendships going on. I mean there’s plenty of groups of men I’m sure who have been together for a long time you know, at various intervals of time and all they do is just get smashed out of their brain whenever they meet up, so I’m not saying it happens in all... But, in that type of friendship group where there’s genuine concern, yeah I don’t think you’re not going to turn up one day and find that someone is blubbing with his head on the table and you’ve got to put your arm around him and say “there, there, what’s happening” but, similar feelings could well be churning around inside him but, it’s going to take a fair bit of skill (laughing) to try and extract something and get it sort of out in the open and getting it ventilated, talked about or whatever might be useful.

As we can see, with Fabien and his friends there is an ethic of care (*“keep an eye on each other”*; *“genuine concern”* etc.) notwithstanding the “covert” ways this may be conveyed and the “skill” that may be required to elicit disclosures. Clearly, it is important to stress the ability of peers to recognise when someone is in distress and to facilitate emotional talk. Sometimes participants distinguished between who and when they might open up, for example:

If I was at university and dating, I would, you know, seek to speak to like my best friends first um, I would actively seek them out. However, if I was like walking back from a lecture with a friend... and it would just let slip. I haven’t seeked them out but I’m with them now and I’m going to say it... you kind of keep it a bit vague rather than being like, with a best friend, you’d kind of like divulge “Oh I’m really into this girl” but with them, you’d go “oh yeah, you know, I’ve sort of been seeing ...” (laughing) and then you see what their reaction is and they’re probably doing the same thing. They’re probably a bit guarded. Thomas (P2)

So, engaging in emotional talk with “friends” can be potentially risky, and in remaining vague and potentially downplaying the emotional nature of the conversation, men may be able to navigate constraints on men’s emotional talking practices. Moreover, in highlighting that he would *“see what their reaction is”*, the importance of the other in social encounters is reiterated. The role of other individuals in interpreting covert forms of communication and in encouraging and facilitating more direct conversational styles is particularly important here.

Some participants from phase 3 engaged with direct, emotional talking practices continuously with little regard for gendered “norms”, while for other participants the use of open talking practices remained context-dependent. With a changing climate toward mental health and well-being in the United Kingdom generally, especially with a growing recognition of mental health in the emergency services, Leo (a firefighter) has been able to establish a safe space at his work:

Without trying to toot my own trumpet, I’ve been quite big and open about it. I’ve done videos that have gone round the brigade and written blogs and done all sorts of things um, that have sort of put me on the chopping block and actually the response has been pretty positive from a lot of people... we’ve had some pretty crap events... two people commit suicide in the last couple of years through mental ill-health, so it’s almost been forced on us as something that this isn’t something you can ignore, you know, there’s serious consequences.

By reframing speaking out about his mental health issues as “quite big”, Leo’s has been able to “strategically negotiate hegemonic norms … to fashion a more “positive’ masculine performance” (Lomas, 2013:178). This ability of men to re-negotiate traditional ‘norms’ into more positive forms of behaviour has similarly been documented in an array of social and health contexts (see De Visser et al., 2009; Sloan et al., 2009).

For Ralph (P3), who has gone through counseling and has used mindfulness to help him manage his anxiety, talking to others became important:

There were a change in me... and I thought well, I know what I’m talking about yeah, you know, and I thought well, you know what’s the worst that can happen... If you’ve got mental health issues, then you’ll talk to your girlfriend about it where men won’t. There’s still that, that alpha male thing where you won't say. I mean, I didn’t know until I got to the point I got to and I were talking to a couple of me mates and they’d been suffering from depression for years and they’d never mentioned it and I’ve never mentioned mine.

Ralph positions himself as an experiential expert and qualified to speak with authority on anxiety following his mental health journey and engagement with services. Again, the female partner is invoked as a safe harbour where feelings can be shared while other men are (again) construed as reticent. Taking the first step to talking openly in all-male social settings may be particularly daunting-a sentiment that was echoed by Dylan, who argued *“The hardest step for a man is the first time they talk about it and speaking out”*. However, it appears that taking the first step in an all-male setting can facilitate emotional engagements from other men. Dylan’s experiences highlighted the power of “the first step”, for his own confidence in emotional communication and in encouraging him to engage other men. Reflecting on how he initially began to work in mental health activism, Dylan recalled:

I was invited to an event in the museum one night... came out me flat, pouring with rain, freezing cold wind but I thought “go on, make an effort and go along”. Sitting at a table by myself with a glass of orange juice-I mean there’s an achievement, glass of orange juice (laughing) and tap on the shoulder, I looked around all I could see was a pair of kneecaps. This guy is about 6 ft. 6. Introduced himself as Toby, a service-user lead... I told him about myself and what I’d been through, showed him me arms... and he was one of the first people in my life that had never judged us.

In presenting himself as a service-user and initiating a conversation about mental health, Toby was able to create a “safe space” for Dylan to talk about his struggles. He established shared ground and maintained a non-judgemental approach that was different from Dylan’s past experiences with other men. Establishing shared ground between men may be particularly transformational in facilitating men’s engagements with emotional talk. The approach that Toby took toward Dylan when they first met is one that Dylan now uses to approach other men who may need help:

A guy came along here a few months ago. First, he wouldn’t speak to us and I thought well I’ll tell him about my background and my history and then he kind of trusted me a lot more... He’s coming along to training and stuff as well and he said to me, had I been a psychiatrist or doctor, he would’ve just backed off.

Again, we can see the importance of establishing trust in encounters in which men are expected or required to engage in emotional topics or conversational styles. The establishment of trust in working with men in health contexts has been highlighted as particularly important in facilitating engagements with health professionals and in encouraging repeated engagements (Robertson et al., 2015). Establishing shared ground and trust within an all-male social group may be an effective way to encourage men to speak more directly and openly about their emotions and mental wellbeing. Research on men’s use of online forums to discuss sensitive issues has also suggested trust as an important factor in men’s delayed help-seeking in face-to-face contexts and as a facilitator of men’s disclosure and discussion of a range of topics, from depression (Gough, 2016) and anxiety Drioli-Phillips et al. (2020a), Drioli-Phillips et al. (2020b), to body image and eating disorders (Flynn and Stana, 2012). Indeed, it appears that establishing trust or “safe spaces” may be vital in a range of contexts, both homosocial and otherwise, to encourage men to engage with emotional topics and to disclose any mental health and wellbeing concerns.

## Discussion

Our analysis highlights that men’s anxiety-talk and help-seeking is embedded in (and often constrained by) interpersonal relationships and social interactions, and in wider discourses around masculinities. The impact that close romantic relationships can have on men’s emotional talking practices was especially clear. The majority of the participants who were married or in long-term committed relationships understood emotional intimacy and expression to be a core tenet of a strong, sustainable relationship–notwithstanding some tensions and complexities at times. With male friends and acquaintances, participants reported being wary of self-disclosure and only volunteered information indirectly, if at all, depending on the nature of the relationship/s and social context. Overall, the importance of trust and establishing shared ground between speakers was foregrounded. To echo Lomas (2013: 177), “restrictive emotionality is not inevitable in men … when men are given permission and safety to talk, they are well capable of insightfully analysing and sharing their emotions”. What is clear is that men do talk, in a range of ways and for a variety of reasons. However, an often-overlooked aspect of men’s emotional talking practices relates to the ability of others to recognise and encourage these practices. Thus, while the answer to the question “do men talk about their emotions?” is a resounding yes, one further, important, question remains: are we listening?

Despite the overwhelming emphasis placed on individualistic approaches to mental well-being and recovery, smaller bodies of research have highlighted that recovery in mental health terms is an “inherently social process” (Marino, 2015). Indeed, qualitative research highlights the crucial roles that family members, friends, professionals and an individual’s broader community play in facilitating recovery from mental illness (e.g., Price-Robertson et al., 2017). While the key role of intimate partner relationships in facilitating men’s emotional communication is well established, more research is required into the nature and form of men’s emotional talking practices with other men, including (best) friends but also in the context of emerging community therapeutic groups for men (e.g., https://andysmanclub.co.uk/). Until now, researchers interested in male help-seeking, peer support and self-disclosure have largely been confined to online spaces such as discussion forums dedicated to health and wellbeing issues (e.g., male infertility; men and depression) where contributors are anonymous. Gaining access to offline, face-to-face meetings where men share stories of anxiety, depression and loss could provide additional valuable insights and recommendations for developing further, creative interventions tailored to specific groups of men.

Our analysis chimes with much of the qualitative research on depression which highlights the constraints associated with masculinity norms, the importance of partners and others in facilitating (partial) self-disclosure and help-seeking, and the (restricted) nature of emotional communication between men. Indeed, it must be emphasised that some of our sample are likely to have experienced co-morbid depression (two phase three participants explicitly mentioned a depression diagnosis), not to mention other mental health issues (two cited PTSD), so we must take care not to attribute our findings to anxiety-related issues exclusively. It is also worth drawing attention to the role of mental health literacy-those men who had been through counseling following an anxiety diagnosis were typically more inclined to self-disclose with others, male or female, most likely because of prior experience of doing so in formal contexts. Although men in general are thought to have limited knowledge and skills around emotional communication (see e.g., Ogrodniczuk et al., 2017), most of our participants referenced disclosing anxiety and related issues to others within relationships and contexts in which they felt safe and comfortable. It is clear, though, that many men require greater education and training in relation to mental health literacy (see Levant et al., 2009). In addition, research on men and anxiety is scarce and much more work is required to understand how men in different social positions, cultural locations and community settings communicate and manage their anxieties.

## Data Availability

The datasets presented in this article are not readily available because not publically available. Requests to access the datasets should be directed to b.gough@leedsbeckett.ac.uk.
